# Memorial address for Carlos M. G. Duran, M.D., Ph.D.

**DOI:** 10.1007/s11748-017-0801-1

**Published:** 2017-07-14

**Authors:** Yukikatsu Okada

**Affiliations:** Heart Valve Center, Midori Hospital, Kobe, 1-16 Edayoshi Nishi-ku, Kobe, 651-2133 Japan

**Fig. 1:**
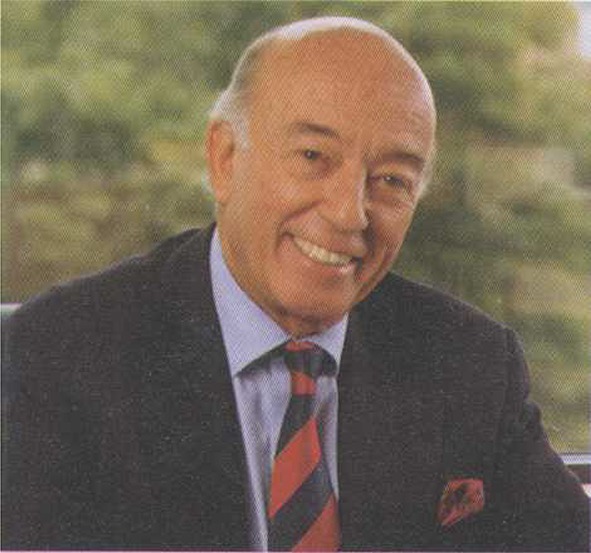
Carlos G. Duran M.D., Ph.D.

Prof. Carlos G. Duran (Fig 1) passed away on his 85th birthday, June 11th. He and his wife had returned to their family home in Bilbao this spring due to his failing health.

Prof. Duran was born in Tetuan, Morocco in 1932 as the son of a Belgian mother and a Spanish father who was a general surgeon. Prof. Duran completed his M.D. at Madrid University in 1956, followed by a residency in General and Thoracic Surgery at the Hopitaux de Paris and then a Ph.D. at Oxford University, with an emphasis on heart valve replacement. His most noteworthy invention was the Duran ring, an annuloplasty system for repair of the mitral and tricuspid valves. He established cardiovascular surgery programs at Navarra University Clinic, Pamplona, Spain, Valdecilla National Medical Center in Santander, Spain, and King Faisal Hospital in Riyadh, Saudi Arabia. He moved to Missoula, Montana, US in 1995 and founded the International Heart Institute of Montana.

He was deeply curious, exceedingly generous, and a true scientist–scholar. To his fellows-in-training, Prof. Duran was known to say “Don’t believe a word I tell you. Challenge everything. This is how medicine advances”.

I met him first at a Mitral Valve Workshop in La Jolla, California in 1988. There were 5 live demonstrations of mitral valve repair and echocardiographic assessment of mitral valve before and after repair. I was very impressed in terms of the teaching style of surgical procedures. I learned “Flip-Over technique” for anterior mitral prolapse there. When I returned to Japan, a 48-year-old lady suffering CHF due to anterior leaflet prolapse was waiting for surgery in the ICU. The patient underwent “Flip-Over technique” with good results. In 1990, I again attended a mitral workshop in Tampa, Florida. There were live demonstrations, lectures, and a wet lab using porcine hearts. Since we had an opportunity to start a clinical trial of the Duran flexible ring in Japan, I learned a lot about size selection and implanting procedure of the Duran flexible ring using a porcine heart.

After clinical application of the Duran flexible ring, echocardiographic analysis of the mitral annulus with the Duran flexible ring allowed us to understand the flexibility of the mitral annulus after repair. I wrote the manuscript and faxed it to Prof. Duran in Saudi Arabia.

The manuscript was revised in 2 days and I had a call from Carlos. He said “What do you think about my revision?” The manuscript entitled “Comparison of the Carpentier and Duran Prosthetic Rings Used in Mitral Reconstruction” was accepted without revision and was published in1995.

**Fig. 2:**
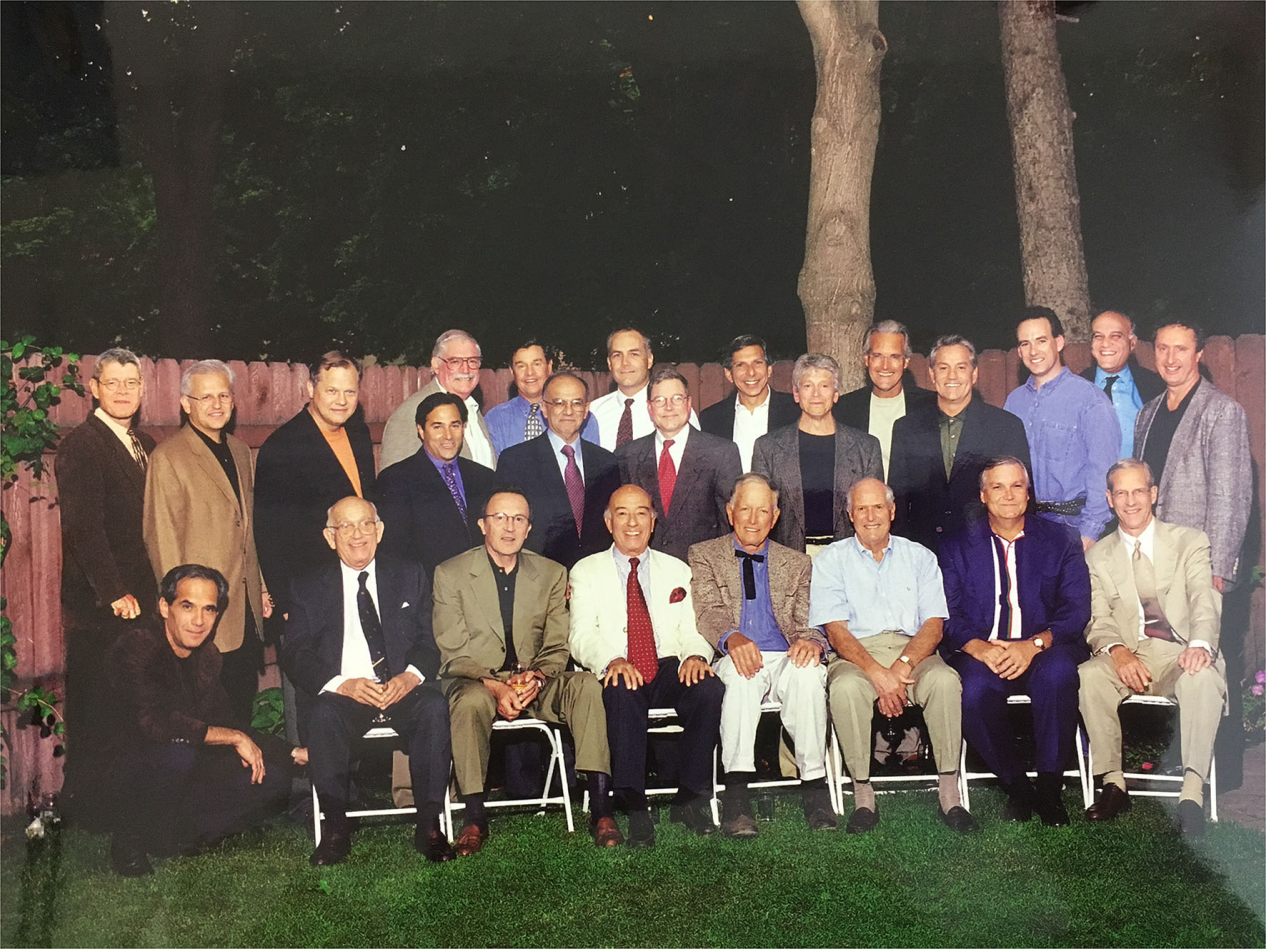
Memorial picture

Prof. Carlos G. Duran and his colleagues, Dr. J. Oury, Dr. P. Shah, Dr. D. Rubenson organized the Rocky Mountain Valve Symposium. The main theme of the Rocky Mountain Valve Symposium included mitral valve repair, tricuspid repair, aortic valve repair, valve sparing technique, Ross procedure. Live surgeries by Prof. D. Ross, Prof. A. Carpentier, Prof. M. Yacoub and Prof. T. David were very impressive. Prof. Duran taught us the contraindication for mitral valve repair (Table 1). As this concept was the start of my mitral valve repair, I have challenged so many reparative techniques. I have attended the Rocky Mountain Valve Symposium 15 times because of attractive ideas for the management of patients with heart valve disease. The picture is the Rocky Mountain Valve Symposium’s 10th anniversary. You can see the giants who made a history of heart valve surgery (Figure 2).

**Table 1:** Contraindications for mitral valve repair

The main limitations to repair mitral valve are
Poor visibility
Heavy and generalized calcification
Severely fibrotic and immobile leaflets
Severely deformed subvalvular apparatus
Active non-localized infection

Prof. Duran mentioned about the educational activity. We cardiac surgeons have a 30-year professional career. At first 10 years, please obtain skills and knowledge about cardiac surgery, then challenge the new procedures and approaches in the next 10 years, and keep educational activities at last 10 years. Educational activity is very important to maintain patients’ life style. Since life span of the patients often exceeds more than 20 years, only good education can retain the life style of the patients.

Prof. Duran always stimulated and encouraged me to consider something new. He always asked me “Yuki, what are you planning for your job now, when do you present your work, when do you publish your work?” I had to prepare something new before meeting him. He listened to my talk very carefully and gave me good advice. He did not ignore or neglect my considerations. There was a funny story concerning chordal cutting for ischemic mitral regurgitation. He completely opposed the idea of chordal cutting in terms of left ventricular function after surgery. When we published chordal cutting for IMR in the *Journal of Cardiology*, he asked me “why do you cut the important strut chordae in terms of LV function?” He, however, gave me an opportunity to present clinical results of chordal cutting for IMR at the Rocky Mountain Valve Symposium. He said “Yuki, please follow the patients very closely”. He did not neglect or ignore the new ideas.

There was another good story in Paris. When I attended a mitral workshop in Paris, Prof. Duran and I incidentally climbed up the stairs. As Carlos kept carry-on luggage, I asked him “May I help you?” His answer was “Yuki, please look around me. You can see so many young girls. I am a man”. I said “I am very sorry. You are a Spanish man”.

Prof. Duran and his colleagues in Missoula, Montana pushed me to start the valve workshop in Japan. Hanshin Heart Valve Symposium started in 2002 in Kobe. Live surgery including mitral valve repair and aortic valve repair, replacement with Freestyle bioprosthesis, lectures and wet labs according to the Rocky style were successfully carried out for 10 years. Echocardiologists were the key members to organize this kind of workshop. Prof. Yoshikawa and his colleagues exclusively supported Hanshin Heart Valve Symposium. Prof. Yoshikawa, Prof. Shah, and Prof. Duran were really close friends. Hanshin Heart Valve Symposium was changed to the Japanese Society for Heart Valve Disease.

In March 2011, the cumulative number of patients who underwent mitral valve repair at Kobe City Medical Center approached 1000 cases. Prof. Duran gave me a message (Table 2). It was a great pleasure for me and our team.

**Table 2:** Message from Carlos G. Duran

Dear Yuki, congratulations for your achievement of 1000 cases of mitral repair. It represents a tremendous amount of work, persistence and faith. It brings wonderful memories of the early cases you did and the gestation of your publications that helped to popularize the repair techniques in Japan. It makes me happy, thankful and proud of having participate in your achievement
On a sad side I am sorry to hear about the earthquake, Tsunami and now the nuclear power plant crisis. What a disaster! Because you are geographically a long way from the disaster area I assume that you and your family are OK. However, it will affect your whole country for quite a long time. Our heart is with you. Carlos

Prof. Duran introduced me to a lot of cardiac surgeons and cardiologists who were interested in the management of the patients with heart valve disease. He used to say “we are colleagues to seek better management of the patients with heart valve disease. Yuki, don’t hesitate to discuss the clinical topics in the surgical treatment, please”. It is my great pleasure to keep friendship with many of them.

Carlos, we will try to inherit your spirit to the next generation. I sincerely appreciate your long-term friendship.

